# Analysis of consumer‐oriented quality characteristics of raw and boiled plantains in Cameroon: implication for breeding

**DOI:** 10.1111/ijfs.14812

**Published:** 2020-10-13

**Authors:** Gérard Ngoh Newilah, Cédric Kendine Vepowo, Annie Takam Ngouno, Alexandre Bouniol, Agnès Rolland‐Sabaté, Vivien Meli Meli, Judéon Socrates Yong Lemoumoum, Lora Forsythe, Dominique Dufour, Geneviève Fliedel

**Affiliations:** ^1^ University of Dschang PO Box 96 Dschang Cameroon; ^2^ Centre Africain de Recherches sur Bananiers et Plantains (CARBAP) PO Box 832 Douala Cameroon; ^3^ CIRAD UMR QUALISUD Montpellier F‐34398 France; ^4^ Qualisud, Univ Montpellier, CIRAD, Montpellier SupAgro, Univ d'Avignon, Univ de La Réunion Montpellier F‐34398 France; ^5^ INRAE UMR SQPOV Avignon Université Avignon 84914 France; ^6^ National Research Institute University of Greenwich Central Avenue Chatham Maritime Kent ME4 4TB UK

**Keywords:** Boiled plantain, Cameroon, focus group discussions, individual interviews, plantain processing, quality characteristics, surveys

## Abstract

This study aimed at understanding users’ demand for raw and boiled plantains in rural and urban areas in West and Littoral regions of Cameroon. Surveys conducted in eight rural localities consisted of key informant interviews, gender‐disaggregated focus group discussions, market and individual interviews. Processing and cooking diagnoses were done with restaurant cooks in urban areas, to know the details of plantain processing and boiling and to understand the quality characteristics of raw plantain that give a most‐liked boiled plantain. Local favourite landraces, most cultivated landraces in Cameroon (*Batard* and *Big ebanga*) and a new CARBAP/CIRAD hybrid (*CARBAP K74*) were used. The preference for plantain cultivars was both gender and region‐dependent. High‐quality plantain should be mature, with big fingers and having a dark green peel colour. The fruit length and girth, pulp pH, dry matter content and firmness were found to be relevant postharvest quality characteristics for plantain breeding improvement.

## Graphical abstract

Users’ demand for raw and boiled plantain in West and Littoral regions of Cameroon are driven by fruit’s length, fruit’s girth, pulp’s dry matter content, pulp’s pH and pulp’s firmness. These are pertinent post‐harvest quality traits for breeders.

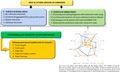

## Introduction

*Musa* spp., including plantain, banana and cooking banana, is a major fruit group in the world. In 2018, production was estimated at about 139 470 376 metric tonnes (MT), with plantains making up 15% (Lescot, 2020). In Cameroon, banana production was estimated at 1 203 440 MT while that of plantain reached 3 940 818 MT (FAOSTAT, 2020). Plantains and other cooking bananas are staple food crops for approximately 70 million inhabitants of West and Central Africa (Pick *et al*., 2000). Preparing plantain and cooking bananas for food uses varies according to country, with main forms being boiled, roasted and fried (Robert *et al*., 2017). In Cameroon, over eleven food uses of plantains and cooking bananas which depend on cultivar and consumption habits were reported (Ngoh Newilah *et al*., 2005, 2018). When boiled, they are either eaten as a main dish with liquid sauces, vegetables, legumes or as an accompaniment to other foods like rice or pasta in order to increase the consistency of the meal. Little is documented on the reasons for choosing plantain cultivars for a particular food use. Tchango Tchango *et al*. (1999) reported that quality characteristics of plantain cultivars may either be physical (colour, length, size, shape and texture) or chemical (total soluble solids, pH and titratable acidity). Moreover, Gibert *et al*. (2009) reported that pulp dry matter and pH helped to discriminate *Musa* varieties, with plantain subgroup exhibiting the highest values (41.1% and 5.6%, respectively). In addition, the specific cooking process of plantain could have an impact on its final quality. It was important to understand the choice criteria used by food chain actors in order to enable breeders to improve hybrids for better adoption and impact.

Fliedel *et al*. (2016) developed a new approach aimed at providing information on selection criteria for quality traits to breeders early in varietal improvement programmes. It involved successive steps including qualitative surveys along the food chain to identify quality criteria of a good cassava crop and product, and effective participation of processors to identify the suitability of new genotypes for a good product.

Forsythe *et al*. (in press) adapted and expanded this approach in an interdisciplinary and participatory methodology to better understand end‐users’ demand of good quality root, tuber and banana (RTB) crops and their food products. The objectives of this study were to use this methodology to identify factors and characteristics that influence cultivar and food product preferences across the food chain for raw and boiled plantain, including growers, processors and consumers.

## Materials and methods

Prior to the implementation of these activities, an ethical clearance was obtained from the Cameroon National Ethical Committee for Research on Human Health. Before each interview, participants signed consent forms.

These activities enabled us to understand the practices and preferences behind boiled plantain along the food chain by answering these questions: what are the different characteristics of the crop? What are the processing and preparation methods that contribute to producing a good quality product? By what factors do these characteristics vary?

This study took place in the West and Littoral regions of Cameroon, using Steps 2 and 3 of the methodology described by Forsythe *et al*. (in press) [this paper is a ‘Registered Reports’ publication type, of different steps developed in 2018. Each step being reported in the manuals (RTBfoods Guidelines) and accessible online]. This methodology aims at determining traits of importance to users along the food chain, in order to develop food product profiles that support breeding and selection decisions. The steps involved in this methodology include:

1. Step 1. Research teams conducted a state of knowledge (SOK) review to establish what is known about the product and the gaps in knowledge in relation to food science, gender and markets in the country context, and to establish the scope of the further studies (Forsythe *et al*., 2018a; https://doi.org/10.18167/agritrop/00568).
2. Step 2. Experts carried out a gendered food mapping exercise in communities to identify the different uses of the crop by different users (e.g. producers, processors, consumers, local retailers) and the associated quality characteristics. The study also investigated gender and market dynamics in relation to the crop and product, and their quality characteristics. At this stage, the first draft of the Food Product Profile containing prioritised quality characteristics by user group is produced, taking into account gender and livelihood context (Forsythe *et al*., 2018b; https://doi.org/10.18167/agritrop/00569).
3. Step 3. Teams conducted a participatory processing diagnosis with experienced processors. Both preferred and non‐preferred varieties were included to provide a wide range of technological and physicochemical characteristics. Processors provided feedback on the varieties before processing, during each processing step, and after processing to identify quality characteristics of the crop and product. Processing parameters were measured at each step. New quality characteristics from this Step are added to the Food Product Profile (Fliedel *et al*., 2018b; https://doi.org/10.18167/agritrop/00570).
4. Step 4. Consumer testing was conducted with approximately 300 consumers in rural and urban areas, to provide a better understanding of consumer demand and to obtain a sensory mapping of the overall liking of each product that could be related to most‐liked and least‐liked characteristics used by each consumer to describe the product. At this stage, new quality characteristics and their prioritisation are added to the Food Product Profile (Fliedel *et al*., 2018a; https://doi.org/10.18167/agritrop/00571).

### Gendered food mapping at the rural level

Inputs from food system users of boiled plantain were received through surveys (Key informants, market and individual interviews) and discussions (sex‐disaggregated focus groups) at the rural level. The purpose was to identify quality characteristics of raw and boiled plantain along the food chain by different stakeholders. This included eight villages: four in the Littoral Region (Sokelle, Song‐mayo, Kombe, and Bouba) and four in the West Region (Bafounda, Bamendjing, Penka‐Michel, and Balessing). They were selected as production areas supplying a large variability of plantain cultivars in major city markets (Douala, Bafoussam and Yaoundé). Moreover, the regions are characterised by two distinct agro‐ecological zones with altitudes ranging from 80 m above sea level (masl) for the Littoral region to 1200 masl for the West region. Two gender‐disaggregated focus group discussions (FGDs), one key informant interview (KII), nine to ten individual interviews (II) and one market interview (MI), were conducted in each village, as described by Forsythe *et al*. (in press). These interviews were complementary and enabled us to obtain extensive relevant information. The interviewed participants worked in diverse activities related to plantain production, processing, trading and consumption. With the help of a community contact, key informants, participants to focus group discussions, to market interviews and to individual interviews were purposively selected based on their involvement in plantain‐related activities (production, processing, consumption, marketing). Where possible, equal numbers of men and women were interviewed in each of the categories. Finally, 110 interviews were administered in French, Pidgin English, vernacular or local language. Two hundred and twenty‐five persons participated to the interviews. For FGDs, men accounted for 51%. For II in the eight villages, thirty‐eight men and forty women participated.

### Participatory processing diagnosis

**Figure 1:**
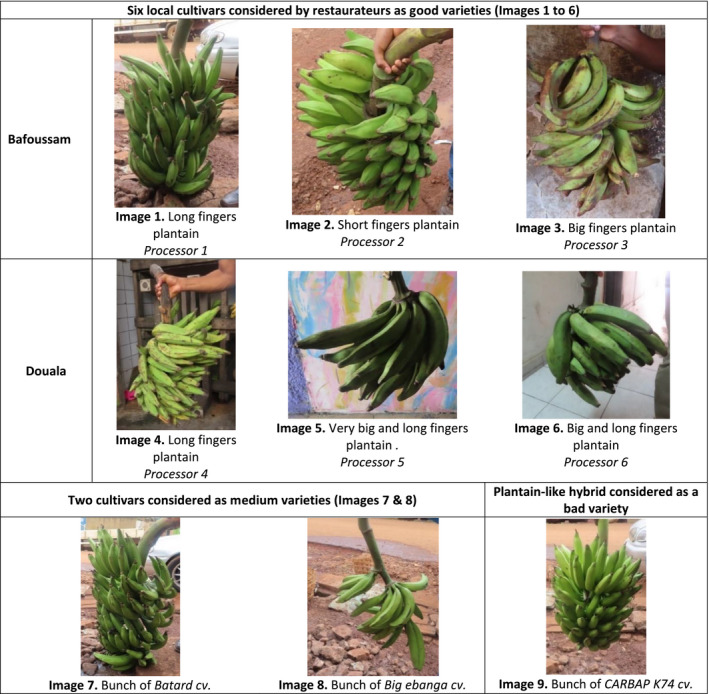
Images of cultivars used by processors in Bafoussam and Douala.

In Douala and Bafoussam, processing diagnoses were carried out with six randomly selected restaurant cooks, following step 3 of the methodology described by Forsythe *et al*. (in press). This helped to assess high‐ and low‐quality characteristics regarding raw and boiled plantains. Cooks were chosen for this study as they are best placed to know what consumers want. Three processors per city participated in the study and chose a working day that suited them. A full day was needed to conduct participatory processing diagnosis with each processor. Processing diagnostics were carried out using four cultivars: (i) a cultivar considered to be good, corresponding to the local cultivar frequently used by the processor in his/her restaurant; (ii) two intermediate quality cultivars (*Batard* and *Big ebanga*), harvested from CARBAP’s experimental plots in Njombé; and (iii) one poor cultivar, *CARBAP K74* (a CARBAP‐CIRAD co‐ownership plantain‐like hybrid created in Cameroon) harvested from CARBAP experimental plot in Bansoa (Fig. 1). Plantain bunches were at their full green stage (unripe), to avoid ripening stage variability during processing diagnosis. This full green maturity stage was assessed by observing the pulp colour, the peel colour, the shape (fullness) and angularity of the fruit. Immature fruit is angular in cross‐sectional shape, has distinct ridges, has cream or white pulp colour and light peel green colour. As the fruit matures, it becomes less angular, more rounded or full, has an orange pulp colour and a dark green peel colour. The degree of roundness differs between cultivars and location of the hand on the bunch. Processors in each restaurant used the kitchen tools proposed by the research team to avoid any other bias.

### Data collection and analysis

After survey data collection, answers were translated into English and transcribed data were transferred to a Microsoft Excel file for thematic coding. Qualitative (e.g. characteristics’ description) and quantitative (e.g. characteristics’ count and priority given) data analyses were performed according to Forsythe *et al*. (2018a, 2018b,2018a, 2018b).

#### Data collection

During participatory processing diagnoses, parameters measured included (i) fruits’ length and girth using a measuring tape; (ii) fruit thickness using a pair of callipers; (iii) weight of plantain pulps before and after cooking using a balance load‐cell. Pulp to peel ratio percentage (P%) was recorded by weight difference as follows: P% = [100 × (FW − PW):FW] where FW and PW are weights (g) of the fruit and peel, respectively. The quantity of water absorbed by the plantain pulps during cooking was calculated as the weight gain; (iv) fruit peeling time, pulp scraping time and cooking time of pulp or fruit. Cooking time refers to the time between which the pot (containing water) is set on the fire with the pulps or with unpeeled fruit until cooking is completed, measured using a stopwatch and expressed either in seconds (s) or minutes (min); (v) quantity of water used for boiling, measured with a 2000 mL measuring cylinder at the start of the demonstration; (vi) boiling temperature, measured using a kitchen thermometer once the water was boiling, and expressed in degrees Celsius (°C); (vii) pulp firmness, assessed with a handheld force gauge (PCE‐FM200) on raw and boiled plantain pulps that were cut transversely at their midpoint and placed on a flat surface. The force required to penetrate 1 cm of pulp tissue with a 6 mm diameter cylindrical probe was measured and expressed in kilogram force; and (viii) overall preferences of boiled plantain of four cultivars from each processor in each town using simple ranking.

Samples of raw and boiled plantain pulps were collected at each restaurant and analysed for total titratable acidity (TTA), dry matter contents and pH, according to Dadzie & Orchard (1997).

#### Statistical analyses of data

XLSTAT 2014 Software was used to analyse all the data. Results are presented as means ± standard deviation. Statistical analyses were done on the results using one‐way anova to determine the significant differences in the values at *P* < 0.05 (Student Newman Keuls’ Test).

Data Normalisation and Graphical Combination: The most relevant parameters highlighted by statistical analysis were selected. The parameters were individually normalised in the interval 0–1 (where 0 and 1 represent the smallest and highest value respectively for each parameter) combining data of various cultivars used in this study. The normalised data were averaged per cultivar prior to being graphically combined within a radar chart.

## Results and discussion

### Quality characteristics of plantain for boiling

#### Preferred cultivars

Plantain cultivars that give the highest quality of boiled plantain differed by region, but women and men preferred the same cultivar (Ebanga), which has different names according to women in FGDs. Women specifically preferred ‘big plantain bunch with big fingers’, characterised by its lax bunch (one in which one can easily place a hand between the hands of the fruit) with 8–10 hands and fruits as long as 31 cm. In the West region, ‘Kendon netôh’ was the best cultivar, with its lax bunch, composed of 4–6 hands, and big fruits of 28–30 cm long. In the Littoral region, ‘Ebang’ was the favourite. This cultivar has also a lax bunch as Ebanga, but with shorter fingers (25 cm) and a reduced number of hands (7–9). Table 1 highlights the first five cultivars that give the highest quality boiled plantain in both regions and for both genders. According to Ngoh Newilah *et al*. (2005), the names of these cultivars may differ among ethnic groups and families. Thus, the designation of a cultivar by its description such as ‘big plantain bunch with big fingers’ connotes that the population is sometimes not aware of the name of the cultivar they planted.

**Table 1:** Plantain cultivars or description of cultivars (*in italics*) with the highest quality boiled plantain (data obtained from FGDs)

Importance	Women	Men	West region	Littoral region
1	*Big plantain bunch with big fingers*	Ebanga	Kendon netôh	Ebang
2	Ebang	Big Ebanga	*Big plantain bunch with big fingers*	Ebanga
3	Ebanga	Ebang	Fon ngwô*	Nkounda^†^
4	Kendon netôh	*Plantain bunch with small fingers*	*Plantain bunch with small fingers*	Brokaka^‡^
5	Yong^¶^	*Plantain bunch with big fingers*	Mékih tôh^§^	Yong^¶^

* Also called Kendon netôh depending on the locality.

^†^ Plantain cultivar with a compact bunch, short fruits of 22 and 24 cm long, and 7–9 hands.

^‡^ Plantain cultivar with a compact bunch, fruits of 23 and 25 cm long, and 8–10 hands.

^§^ Plantain cultivar with a compact bunch, short fruits of 17 and 19 cm long, and 6–8 hands.

^¶^ Plantain cultivar with a compact bunch, long fruits of 26 and 28 cm long, and 7–9 hands.

#### Raw plantain and boiled plantain characteristics

The most important characteristics of raw plantains obtained from II after simple ranking and required to obtain high‐quality boiled plantains, varied according to gender and region. Men liked plantains whose peels were dark green coloured, with big fingers, while women preferred ‘mature plantains’, with big fingers and dark green coloured peels. People from West and Littoral regions gave similar characteristics, but with different priorities. While people from the West region gave priority to plantain with big fingers, those from the Littoral instead prioritised plantains with a dark green peel colour. In addition, ‘presence of a split finger’, ‘attractive bunch’, ‘absence of sap flow from the fruit’ or ‘orange pulp colour’ (observed when the fruit is broken into two pieces) were also mentioned as important for a high‐quality product in both regions. Dadzie & Orchard (1997) identified fruit diameter, length, bunch age and colour of the peel as some of the external factors for determining the maturity of plantain. Other maturity determining factors also include fruit shape, diameter and length, presence or absence of distinct fruit ridges, fruit firmness and peel to pulp ratio. Immature plantain fruits have more distinct ridges and are less rounded compared to the mature ones. In the same context, Ngoh Newilah *et al*. (2010) reported that the plantain cultivar ‘French sombre*’* is 21 cm long, a grade of 3.5 cm and an orange pulp colour at optimal maturity. Similarly, Ferris *et al*. (1999) presented fruit lengths for plantain hybrids and cultivars varying between 18 and 25 cm, with fruit girths between 12 and 13 cm.

Information obtained from II, and backed up by findings from FGDs, revealed that ‘texture’ and ‘appearance’ are important characteristics for high‐quality boiled plantains. Irrespective of regions and gender, the characteristic ‘soft plantain’ was the most important. Women added that their plantain should be ‘well‐cooked’, besides being soft. Other respondents rather insisted on the colour of the boiled plantain, which should either be ‘yellow’ or ‘brown’. These findings align with those of Qi *et al*. (2000) who reported that texture is viewed by consumers as one of the most important attributes of the cooked product in determining a good banana or plantain. However, some plantain pulps become hard when cooling, probably due to the starch properties, including retrogradation known to occur during cooling (Wang *et al*., 2015), thereby depreciating their quality.

#### Profiling of gender‐related preferences

**Figure 2:**
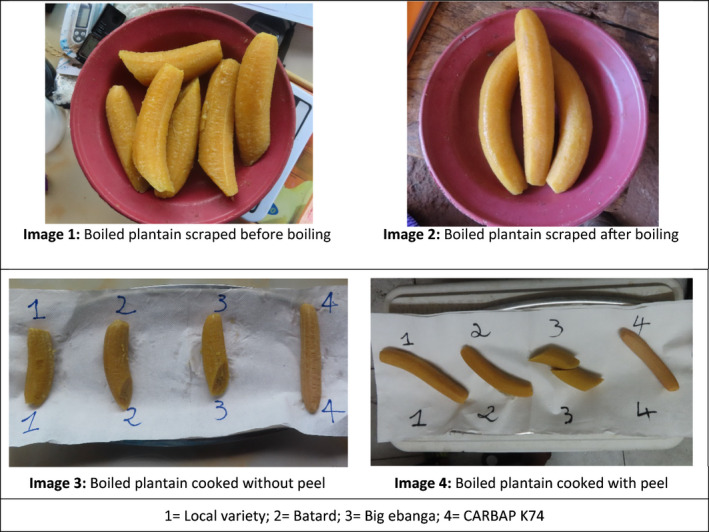
Images of boiled plantain pulps.

Analyses of data from FGDs and IIs permitted to sort out characteristics for both men and women, regarding raw and boiled plantains (Table 2). Men and women stated similar preferred characteristics for raw or boiled plantain. In general, good quality raw plantain should be mature, with a dark green peel colour and big fruits. In addition, the pulp colour should be yellow or orange. The reverse is true for low‐quality raw plantains which are usually immature fruits that are soft on touching, with a light green peel colour. When plantain is boiled, it should be soft in the mouth, well‐cooked, with a yellow or brown pulp colour (Fig. 2). Women added that their boiled plantains should also be attractive.

**Table 2:** High‐ and low‐quality characteristics (in decreasing order of importance) of raw and boiled plantain for men and women

	Raw material		Processing characteristics	Final product	
	High‐quality characteristics	Low‐quality characteristics		High‐quality characteristics	Low‐quality characteristics
Men	Dark green peel colour Bunch with big fruits Yellow pulp colour Mature fruits	Immature bunch White pulp colour Small fruits Light green peel colour Fruits soft (on touching)	Dark green peel colour Bunch with big fruits Mature fruits	Soft pulp Yellow or brown pulp colour Well‐cooked plantain	Poorly cooked plantain Hard plantain Plantain with a poor taste
Women	Mature fruits Bunch with big fruits Dark green peel colour Hard fruits (on touching) Orange pulp colour	Immature bunch White pulp colour Fruits not hard (on touching) Light green peel colour Small fruits	Mature fruits Bunch with big fruits Dark green peel colour Hard fruits (on touching) Orange pulp colour	Soft plantain Attractive plantain Yellow or brown pulp colour Well‐cooked plantain	Poorly cooked plantain Hard plantain Plantain with a poor taste

### Processing raw plantain into boiled plantain

**Figure 3:**
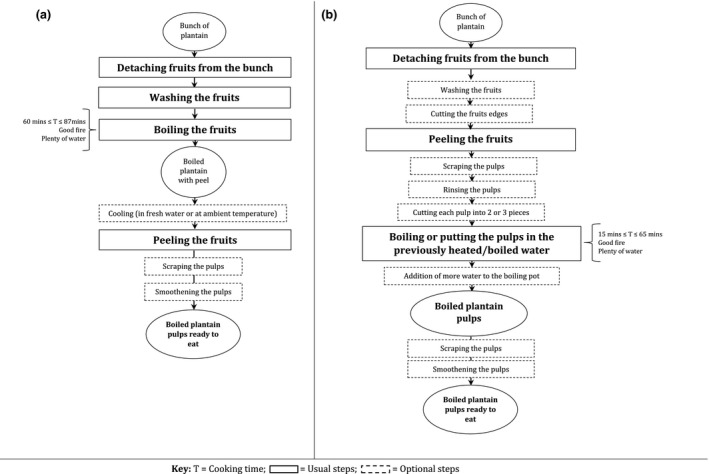
Flowcharts of boiled plantain pulps, when boiling unripe plantain with peel (a) or without peel (b).

The main steps for making boiled plantains are peeling, washing, scraping and boiling. The order may vary depending upon whether the peeling is done before or after boiling. These cooking patterns, with or without peels, are illustrated in Fig. 3 flowcharts.

When plantain is boiled after peeling, processors (restaurant cooks who cook plantain in different forms) either initially heated or boiled the cooking water before introducing the peeled fruits. Scraping consists of removing the remnants of the peeling process on the pulp, alongside the tiny layer that covers the plantain pulps. This is done with a sharp instrument (knife or Indian bamboo) and makes the plantain look more attractive after cooking. When the fruits are peeled, scraping is done either before or after boiling. Meanwhile, when the fruits are not peeled, it is necessarily done after boiling and peeling (Fig. 2: images 10 and 11). The end of the boiling process was mostly assessed either by the change in colour of the peeled fruits or by the splitting of the peels on the unpeeled fruits. Only one processor in Douala cooked her plantains with peels.

### Morphological characteristics of raw plantain

Plantain cultivars used during processing diagnoses were heterogeneous. They presented fruit length ranging between 18.2 and 30.8 cm, with CARBAP K74 and *Big ebanga*, respectively, the shortest and longest. The same observation was made with fruit girth, which varied between 11 and 16 cm. A similar trend was observed for peel thickness, which varied between 3.2 and 4.4 mm. Peel thickness gives an idea of how easy it is to peel a fruit: the greater the peel thickness the easier the peeling. In this respect, CARBAP K74 will be difficult to peel compared to other varieties. Ngoh Newilah *et al*. (2011) reported that in Cameroon, fruit girth is an important criterion for householders during selection of plantain cultivars for specific uses.

### Physicochemical characteristics of raw and boiled plantain

#### Dry matter contents

Dry matter contents (DMC) of raw and boiled plantain pulps varied according to the cultivars and the processors (Table 3). Raw or cooked Batard pulps presented the highest value while CARBAP K74 exhibited the lowest value. Data obtained on raw pulps (an average of 30.0–41.7% w.b) corroborate those obtained by Ngoh Newilah *et al*. (2010) who reported values ranging between 37.8% and 39.4% DMC of raw pulps of French sombre and Pelipita. Similarly, Ferris *et al*. (1999) reported values ranging between 35.9% and 36.2% DMC for plantain landraces, and between 31.6% and 35.1% DMC for plantain hybrids, while Gibert *et al*. (2009) reported an approximate value of 41.1% DMC for raw plantains. The boiled pulps ranged from 27.9% to 39.6% DMC. These results, especially those obtained with local cultivars and Big Ebanga, align with data found by Ngoh Newilah *et al*. (2018), who reported DMC of 37.7% for boiled plantain pulps. Similar results were obtained by Gibert *et al*. (2010) who obtained DMC greater than 32% in cooking bananas.

**Table 3:** Morphological and physicochemical characteristics of plantain cultivars used during processing diagnoses

Locality	Cultivars	Physicochemical characteristics						Morphological characteristics			
		DMC_rp (%)	DMC_cp (%)	F_rp (kgf)	F_cp (kgf)	pH_rp	pH_cp	FL (cm)	FG (cm)	FT (mm)	PT (mm)
Bafoussam	Local cultivar	39.7 ± 1.9^ab^	35.7 ± 2.6^a^	5.4 ± 2.8^a^	2.8 ± 1.3^a^	6.1 ± 0.2^ab^	5.9 ± 0.0^a^	23.0 ± 4.4^b^	14.1 ± 0.6^b^	36.4 ± 2.7^c^	3.9 ± 0.9^abc^
	Batard	41.7 ± 0.0^a^	39.6 ± 0.7^a^	4.2 ± 0.0^b^	2.6 ± 0.8^a^	6.0 ± 0.0^b^	5.7 ± 0.0^ab^	29.0 ± 1.0^a^	14.1 ± 0.3^b^	40.4 ± 0.0^ab^	3.5 ± 0.0^bc^
	Big Ebanga	38.5 ± 0.0^b^	35.6 ± 4.6^a^	4.0 ± 0.0^b^	2.0 ± 1.1^a^	6.0 ± 0.0^ab^	5.8 ± 0.1^ab^	29.5 ± 1.0^a^	14.0 ± 0.6^b^	41.5 ± 0.1^ab^	4.4 ± 0.0^a^
	CARBAP K74	31.8 ± 0.0^c^	27.9 ± 2.1^b^	3.9 ± 0.0^b^	1.7 ± 1.0^a^	6.2 ± 0.0^a^	5.7 ± 0.1^ab^	20.0 ± 1.0^bc^	12.4 ± 0.4^c^	37.4 ± 0.1^c^	3.2 ± 0.1^c^
Douala	Local cultivar	39.4 ± 2.1^ab^	37.0 ± 1.0^a^	5.4 ± 0.2^a^	2.9 ± 0.6^a^	6.0 ± 0.1^ab^	5.8 ± 0.1^ab^	23.9 ± 1.7^b^	13.0 ± 0.7^c^	35.1 ± 2.9^c^	4.0 ± 0.3^abc^
	Batard	40.9 ± 0.0^ab^	38.4 ± 3.9^a^	5.4 ± 0.0^a^	2.9 ± 0.5^a^	6.1 ± 0.0^ab^	5.8 ± 0.1^ab^	22.2 ± 1.1^b^	14.3 ± 0.1^b^	39.9 ± 0.1^b^	3.2 ± 0.0^c^
	Big Ebanga	39.1 ± 0.0^b^	38.2 ± 1.0^a^	4.7 ± 0.0^ab^	3.3 ± 0.7^a^	6.1 ± 0.0^ab^	5.7 ± 0.1^ab^	30.8 ± 0.5^a^	16.0 ± 0.1^a^	43.2 ± 0.1^a^	4.1 ± 0.0^ab^
	CARBAP K74	30.0 ± 0.0^d^	31.0 ± 2.2^b^	4.7 ± 0.0^ab^	1.2 ± 0.6^a^	5.8 ± 0.0^c^	5.7 ± 0.0^b^	18.2 ± 0.6^c^	11.5 ± 0.2^d^	30.0 ± 0.1^d^	3.2 ± 0.1^c^

DMC_cp, dry matter content of cooked pulp (%); DMC_rp, dry matter content of raw pulp (%); F_cp, firmness of cooked pulp (kgf); F_rp, firmness of raw pulp (kgf); FG, fruit girth (cm); FL, fruit length (cm); FT, fruit thickness (mm); pH_cp, pH of cooked pulp; pH_rp, pH of raw pulp; PT, peel thickness (mm).

Means ± standard deviation are data obtained from three replications values with the same letters in the same column are not significantly different at *P* < 0.05 (Student Newman Keuls’ test).

#### Pulp firmness

The firmness of raw and cooked plantain pulps varied for each cultivar. Cooked pulps had a lower firmness compared to raw pulps, no matter the cultivars, with CARBAP K74 presenting the lowest cooked pulp firmness (Table 3). These are in accordance with previous results reported by Gibert *et al*. (2010) for various genotypes of bananas and plantains, even though their values were lower. The higher values observed in this study could be attributed to the equipment used in the determination of the firmness. Gibert *et al*. (2010) measured their firmness using TAxT2 texture analyser platform and revealed that cylindrical borers were not suitable for texture determination during cooking, and as such used instead conical probes. Meanwhile in this study, a cylindrical probe of diameter 6 mm was used for this measurement with a handheld force gauge, contributing to the discrepancies observed. The reasons behind these changes in pulp firmness are numerous, complex and not well known. According to Mohapatra *et al*. (2009), during cooking, insoluble dietary fibre is converted into soluble dietary fibre. Some authors have shown that absorption of water by cellulose, starch, and pectin in the plantain pulp during boiling induces tissue softening (Dadzie & Orchard, 1997; Qi *et al*., 2000), and in particular starch gelatinisation (Gibert *et al*., 2010).

#### Pulp pH

The pulp pH varied depending on whether the plantain pulps were cooked or not. Raw pulps had values ranging between 5.8 and 6.2. Similar values were obtained by Ngoh Newilah *et al*. (2011), Gibert *et al*. (2009) and Passo Tsamo *et al*. (2014) ranging between 5.2 and 6.2. Meanwhile, cooked pulps ranged from 5.7 to 5.9, with CARBAP K74 presenting the lowest values in both cases.

### Synthesis of the morphological and physicochemical characteristics

**Figure 4:**
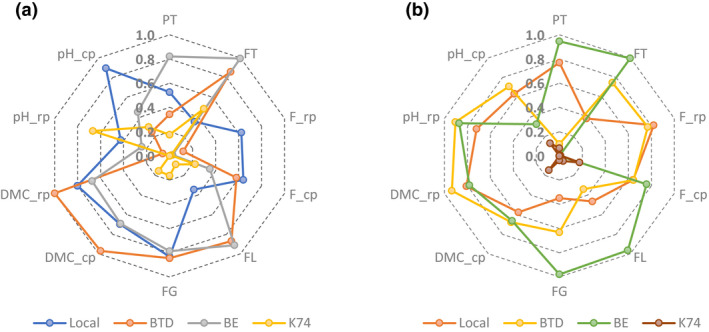
Radar charts of the normalized morphological and physicochemical characteristics of plantain cultivars in Bafoussam (a) and Douala (b). BE, Big Ebanga cultivar; BTD, Batard cultivar; DMC_cp, dry matter content of cooked pulp (%); DMC_rp, dry matter content of raw pulp (%); F_cp, firmness of cooked pulp (kgf); F_rp, firmness of raw pulp (kgf); FG, fruit girth (cm); FL, fruit length (cm); FT, fruit thickness (mm); K74, CARBAP K74 hybrid; Local, local cultivar; pH_cp, pH of cooked pulp; pH_rp, pH of raw pulp; PT, peel thickness (mm).

The synthesis of the morphological and physicochemical characteristics of the various cultivars used by the processors is highlighted within the normalised radar chart (Fig. 4). On the one hand, parameters such as dry matter (cooked and raw pulp), fruit girth, fruit length, firmness (cooked and raw pulp) and peel thickness were higher for plantain cultivars compared to plantain‐like hybrid (CARBAP K74) irrespective of the towns. On the other hand, pH of raw CARBAP K74 pulp was higher than of plantain pulps in Bafoussam. These characteristics therefore suggest that CARBAP K74, compared to the other plantain cultivars, is not a good candidate for boiled plantain.

In order to have a better understanding of consumer preferences, it would be relevant to characterise these *Musa* cultivars using thermal and functional differentiation as done by Dufour *et al*. (2009) and Gibert *et al*. (2013).

### Cultivar preferences and boiled plantain quality characteristics

Table 4 gives overall preferences of the processors for the ability of the cultivars to make good boiled plantain. They preferred Batard and Big ebanga, as these cultivars had the same score (17). However, the occurrence of Batard at the first position (ranked first by three processors) was mainly because it is firm, solid, slender and has a good taste. According to almost all the processors, CARBAP K74 was the least preferred cultivar, because ‘it is like dessert banana and has nothing to do with plantain’. Images 12 and 13 highlight the visual differences existing between these cultivars. However, this plantain‐like hybrid was appreciated by one processor due to its soft consistency in the mouth.

**Table 4:** Overall preference rankings of plantain cultivars by different processors in Bafoussam and Douala

	Preference rankings per processor (1–4)*							
	Bafoussam			Douala			Sum of individual scores (1 = 4, 2 = 3, 3 = 2, 4 = 1)	Overall score (/20)
	P1	P2	P3	P4	P5	P6		
Local cultivar	2	3	3	3	3	1	3 + 2 + 2 + 2 + 2 + 4	15
Batard	1	1	4	1	2	2	4 + 4 + 1 + 4 + 3 + 3	17
Big Ebanga	3	2	2	2	1	3	2 + 3 + 3 + 3 + 4 + 2	17
CARBAP K74	4	4	1	4	4	4	1 + 1 + 4 + 1 + 1 + 1	9

P1–P6 = processors in each town (Bafoussam and Douala).

* 1 = most preferred and 4 = least preferred.

The quality characteristics of the plantain cultivars selected for the participatory processing diagnosis were identified for both raw and cooked plantain by the processors in both towns (Table 5). Maturity of plantain fruits, yellowish pulp colour and long fingers were recorded as preferred quality characteristic at harvest or when buying the plantain bunch. However, when plantain fruits were immature, having low pulp to peel ratio and spotted, they were deemed bad for processing into boiled plantain. Upon boiling, processors claimed that their clients like plantains that are soft, with a natural plantain taste. Besides, they should be attractive, bright and look like ripe plantain. When boiled plantain is sticky, hard, dry or with a taste of sap, then it is of poor quality and would not be appreciated by consumers.

**Table 5:** List of quality characteristics of raw and boiled plantain identified by cooks during processing diagnoses

	Quality characteristics of raw plantain at harvest/when buying	Quality characteristics of boiled plantain			
		Appearance	Texture between fingers	Texture in mouth	Taste	
List of the most‐liked characteristics	Mature plantain fruits Plantains with yellowish pulp Plantains with long fruits	Presentable Bright Attractive Like ripe plantain Thin and nice Yellowish colour Smooth	Soft Supple Tender Wet Mealy	Soft Firm	Plantain taste Natural Good taste Generous	
List of the least liked characteristics	Immature plantain fruits Spotted plantain fruits Plantain fruits with small flesh (fruits with more peel than pulp)	Immature Red colour White colour Pale colour	Sticky Dry Hard	Hard	Banana taste Taste of sap	

### Technological parameters measured during processing of raw plantain into boiled plantain

#### Fruit peeling and pulp scraping times

Fruit peeling was easily done for all the cultivars, except for CARBAP K74 which was deemed difficult to peel by some processors, despite its relatively short peeling times. Scraping the pulp of these fruits depends on the fruit length, with longer scraping times for longer fruits compared to shorter fruits (Table 6). Big Ebanga and Batard showed longer peeling and scraping times, irrespective of the processors or the towns. These cultivars present longer fruits compared to others. For the third processor in Douala who cooked the entire fruit (boiling unpeeled fruit), removal of the peel was easier and peeling times was shorter, while scraping the pulps was longer.

**Table 6:** Some technological parameters on raw and boiled pulps of plantain cultivars per locality

Locality	Cultivars	Peeling time (s)	Scraping time (s)	Cooking time (s)	Pulp %	% Loss of firmness	TTA of raw pulp	TTA of cooked pulp
Bafoussam	Local cultivar	25.43 ± 13.70^a^	34.65 ± 7.79^ab^	2680.00 ± 1173.00^a^	44.88 ± 6.85^a^	50.69 ± 15.91^a^	711.11 ± 138.78^bc^	977.78 ± 167.77^a^
	Batard	25.51 ± 8.09^a^	37.34 ± 21.76^ab^	2620.00 ± 1148.39^a^	49.83 ± 6.23^a^	37.41 ± 18.60^a^	866.67 ± 0.00^a^	1022.22 ± 38.49^a^
	Big Ebanga	29.02 ± 17.38^a^	41.50 ± 6.94^a^	3220.00 ± 658.18^a^	52.55 ± 5.26^a^	49.28 ± 28.25^a^	600.00 ± 0.00^c^	933.33 ± 66.67^a^
	CARBAP K74	15.25 ± 2.17^a^	31.15 ± 15.90^a^	3400.00 ± 421.43^a^	52.67 ± 5.03^a^	57.57 ± 25.11^a^	733.33 ± 0.00^bc^	1000.00 ± 66.67^a^
Douala	Local cultivar	8.66 ± 6.32^a^	13.46 ± 3.38^ab^	2620.00 ± 1654.45^a^	45.31 ± 3.13^a^	46.20 ± 8.73^a^	911.11 ± 138.78^a^	888.89 ± 101.84^a^
	Batard	10.08 ± 7.93^a^	13.37 ± 1.40^ab^	2920.00 ± 2173.57^a^	50.74 ± 3.69^a^	45.36 ± 9.17^a^	933.33 ± 0.00^a^	777.78 ± 138.78^a^
	Big Ebanga	9.69 ± 6.62^a^	20.68 ± 3.82^ab^	2780.00 ± 1472.55^a^	51.08 ± 3.15^a^	29.32 ± 15.61^a^	866.67 ± 0.00^ab^	977.78 ± 76.98^a^
	CARBAP K74	8.57 ± 5.63^a^	11.55 ± 2.92^b^	2740.00 ± 1437.92^a^	49.85 ± 3.31^a^	73.46 ± 12.37^a^	600.00 ± 0.00^c^	977.78 ± 38.49^a^

Means ± standard deviation are data obtained from three replications values with the same letters in the same column are not significantly different at *P* < 0.05 (Student Newman Keuls’ test).

#### Cooking time

The cooking time varied from 15 to 87 min, depending on the plantain cultivar and on the processor (Table 6). In Douala, the highest cooking time (60–87 min) was observed with the third processor. This processor cooked plantains with peels which reduced the heat transfer from water to the plantain pulp, thereby making the cooking longer. The cooking times are quite similar to those reported by Ngoh Newilah *et al*. (2005), who found out that, depending on the cultivar and the ripening stage, the cooking time of plantain ranges between 39 and 87 min.

#### Temperature of water during boiling

Temperature of water during boiling varied from 96.4 °C in Bafoussam to 100.7 °C in Douala. The difference is due to the altitude of these towns, with Douala located at 13 masl and Bafoussam at 1521 masl. As the altitude increases and the atmospheric pressure decreases, the boiling point of water decreases (USDA FSIS, 2011).

#### Weight gain of the boiled plantain pulps

**Figure 5:**
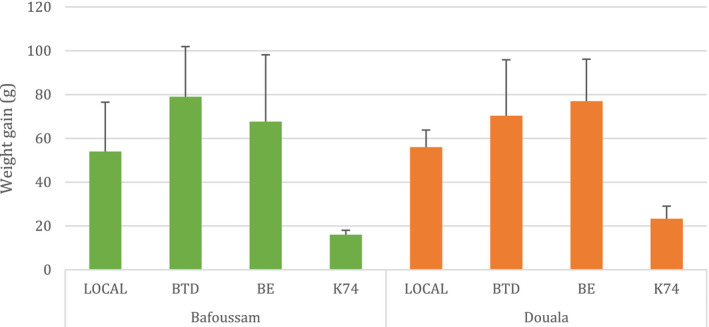
Weight gain of boiled plantain pulps measured after processing diagnoses in Bafoussam and Douala. BE, Big Ebanga cv.; BTD, Batard cv.; K74, CARBAP K74; LOCAL, local variety. [Colour figure can be viewed at wileyonlinelibrary.com]

The weight gain of plantain pulps after boiling is displayed in Fig. 5. Big Ebanga and Batard pulps absorbed more water during boiling than the others, and in particular the local one. CARBAP K74 had the least weight gain during boiling. The relatively low amount of water absorbed by CARBAP K74 is probably due to the genotype functioning differentially to the other varieties. One should note that CARBAP K74 is considered as a plantain‐like hybrid by breeders, rather than a true plantain considering its low starch content. In agreement with this, Belayneh *et al*. (2014) reported that the amount of water absorbed by some cooking bananas during boiling significantly depended on the boiling duration and the cultivars.

#### Percentage loss of firmness

The percentage loss of firmness calculated as (initial firmness − final firmness/initial firmness)*100 is presented in Table 6. From this table, irrespective of the towns, CARBAP K74 is the cultivar that had the greatest firmness loss, especially in Douala (73.46%), while Batard had the least loss. This could be attributed the fact that CARBAP K74 is less rich in starch compared to other plantain cultivars that are rich in starch and firmer in texture.

#### Total titratable acidity

The TTA of a pulp indicates the amount of acid present in it, and it is usually a function of the maturity stage. Upon cooking, plantain pulps become more acidic, with Batard being the most acidic. These values are greater than those of Ngoh Newilah *et al*. (2011), who had values <600 mEq/100 g for plantains at their unripe stage. Knowing that acid is one of the basic components that influence taste, this parameter may be relevant for understanding consumer preferences.

#### Pulp percentage (%P)

The percentage pulp gives an idea on the peeling yield, thus the greater the percentage, the greater the yield. Plantain cultivars having high %P (Table 6) will be appreciated by householders as such cultivars will be ideal for processing. The greatest %P were recorded by Big ebanga and CARBAP K74 in Bafoussam. These low values could reflect the huge amounts of peelings or parts of the flesh which are cut off during peeling and consequently reducing the amount of edible portion of plantain pulps.

## Conclusions

This study enabled the identification of good plantain characteristics, necessary for breeding programmes. In addition to fruit maturity (orange pulp colour), dark green peel colour and large fruit size, other criteria such as fruit length, fruit girth, peel thickness, pulp colour, dry matter contents and pulp firmness were found to be relevant.

Boiled plantain of good quality should be presentable, colour‐attractive, well‐cooked and soft (both on touching and in the mouth). These parameters which are processor‐dependent, need to be translated to quantitative and qualitative data for breeders through physicochemical, thermal and functional characterisation.

Based on the above, landrace plantains are preferred compared to the plantain‐like hybrid studied. Hence, the creation of new plantain hybrids based mostly on pulp firmness, dry matter and the above‐related parameters provides an efficient means of producing high‐quality fruits suitable for boiled plantain. However, while morphological, physicochemical, thermal and functional characteristics are important in discriminating hybrids from landraces, some characteristics related to sensory acceptability are relevant. Therefore, sensory analyses are also needed to evaluate boiled plantain consumer’s acceptability.

## Conflict of interest

The authors declare no conflict of interest in this work.

## Ethical approval

This study was approved by the Cameroon National Ethical Committee for Research on Human Health. The research team obtained ethical approval prior to the fieldwork. Participants were informed about the study, they could stop the interview at any point, written consent from all the participants to the study was obtained, and the research respected the rules of voluntary participation and anonymity.

## Author contributions

**Gérard Ngoh Newilah:** Conceptualization (lead); data curation (lead); formal analysis (supporting); investigation (lead); methodology (lead); project administration (lead); supervision (supporting); validation (equal); visualization (equal); writing‐original draft (lead); writing‐review & editing (lead). **Cédric Kendine Vepowo:** Conceptualization (supporting); data curation (equal); formal analysis (equal); investigation (equal); methodology (supporting); resources (equal); software (supporting); validation (equal); visualization (equal); writing‐original draft (equal); writing‐review & editing (equal). **Annie Takam Ngouno:** Data curation (supporting); formal analysis (supporting); investigation (supporting); writing‐original draft (supporting). **Alexandre Bouniol:** Formal analysis (supporting); methodology (supporting). **Agnès Rolland‐Sabaté:** Investigation (supporting); writing‐review & editing (equal). **Vivien Meli Meli:** Data curation (supporting); investigation (supporting); writing‐review & editing (supporting). **Judéon Socrates Yong Lemoumoum:** Data curation (supporting); formal analysis (supporting); investigation (equal). **Lora Forsythe:** Methodology (supporting); validation (supporting). **Dominique Dufour:** Funding acquisition (lead); project administration (supporting); resources (lead); supervision (supporting); writing‐original draft (supporting); writing‐review & editing (supporting). **Genevieve Fliedel:** Conceptualization (equal); data curation (supporting); formal analysis (supporting); investigation (supporting); methodology (supporting); supervision (supporting); validation (supporting); writing‐review & editing (supporting).

### Peer Review

The peer review history for this article is available at https://publons.com/publon/10.1111/ijfs.14812.

## Data Availability

The data that support the findings of this study are available from the corresponding author upon reasonable request.
